# Prediction of Automotive Wire Harness Aging Based on CNN-biLSTM-Attention

**DOI:** 10.3390/s25092910

**Published:** 2025-05-04

**Authors:** Kun Xia, Qi Zhu, Qingqing Yuan, Jingxia Wang

**Affiliations:** Department of Electrical Engineering, University of Shanghai for Science and Technology, Shanghai 200093, China; xiakun@usst.edu.cn (K.X.); yuanqq@usst.edu.cn (Q.Y.); jingxiawang@usst.edu.cn (J.W.)

**Keywords:** automotive wiring harness, aging prediction, neural network, safety performance

## Abstract

Under the transition towards electrification and intelligence in modern automotive industry, the health status of low-voltage wiring harnesses directly affects vehicle performance and safety. To address the challenge of predicting performance degradation caused by multi-physics coupling effects during wiring harness aging, this study proposes a CNN-BiLSTM-Attention hybrid neural network model. By capturing voltage, current, and temperature parameters during low-voltage system operation, the model combines CNN’s local feature extraction, BiLSTM’s temporal sequence analysis, and attention mechanisms to predict aging levels. Accelerated aging experiments were conducted to obtain wiring harnesses with different degradation levels from new to 720 h aged states, and a dedicated experimental platform was built for data collection and verification. The results show the system achieves a mean absolute error (MAE) of 0.02806, with 32.50% and 62.06% error reduction compared to LSTM and Random Forest models, respectively, demonstrating effective prediction performance.

## 1. Introduction

With the development of modern automotive electrification and intelligence, low-voltage electrical systems will play a more important role in managing various functions of vehicles, including lighting, information entertainment, safety systems, etc. As a fundamental component of this system, the health status of the wiring harness directly affects the performance and safety of the entire vehicle [1,2]. Modern automotive wiring harnesses consist of two primary categories: power cables for energy transmission and data cables (e.g., CAN bus) for signal communication. These two types of cables exhibit distinct operational requirements and aging mechanisms due to their differing electrical loads and environmental exposures. A typical automotive cable comprises a conductor (e.g., multi-strand copper wires) and an insulating layer (e.g., polyvinyl chloride (PVC), cross-linked polyethylene (XLPE), or silicone rubber). Conductor failures, such as strand breakage due to mechanical vibration or corrosion, may lead to increased resistance and localized heating. Meanwhile, insulation degradation, caused by thermal oxidation, chemical corrosion, or moisture absorption, can result in dielectric loss escalation and short-circuit risks [3,4]. Therefore, predicting the aging of wiring harnesses in low-voltage electrical systems of automobiles is of great significance for ensuring vehicle safety and reliability [5].

Currently, research on wire harness aging prediction mainly focuses on developing more accurate and efficient thermal models and intelligent fuse control strategies [6]. By establishing mathematical models for single or multiple cables to describe their thermal behavior, such as heat conduction equations [7], thermal equivalent circuit models, etc., the temperature distribution and variation laws of cables under different working conditions can be analyzed to prevent damage caused by overheating and extend the service life of cables. In [8], a new analytical method is proposed for calculating the transient axial temperature distribution along a single cable. This method considers the nonlinear temperature dependence of cable parameters and solves the self-consistency problem through iterative methods, which is a major improvement over traditional numerical methods and provides a more efficient computational basis for subsequent research. In [9], a method for calculating the thermal resistance of the filling layer using temperature field was proposed for three core cables, and the thermal circuit model was improved to more accurately calculate the temperature of the cable conductor. In [10], the dynamic thermal behavior of high-voltage cable insulation was analyzed, and the transient thermal model of cable insulation was improved by combining theoretical methods and finite element analysis (FEA) methods.

In the field of fault detection and diagnosis of power cables, neural network technology has been widely applied in recent years [11,12,13]. In [14], a partial discharge pattern recognition method based on time-frequency multi view time series (TFMT) model was proposed to more accurately locate partial discharge phenomena in high-voltage cables. In [15], a cable fault prevention method based on multi-valued neural network (MLMVN) and power line communication technology is proposed, which detects cable overheating by monitoring changes in high-frequency signals. In [16], a short-term temperature prediction method for cable joints based on a time convolutional neural network (TCN) and an improved BP neural network is proposed, which introduces a working index variable and effectively improves the prediction accuracy. In [17], discrete wavelet transform (DWT) and chaotic system were combined with a convolutional neural network (CNN) for insulation fault diagnosis of XLPE power cables. By using dynamic error scatter plots as features, fast identification of four different types of faults was achieved.

This article proposes a new method for predicting the aging of automotive wiring harnesses based on CNN-biLSTM-Attention neural network, targeting the data characteristics during the operation of automotive wiring harnesses. Convolutional neural networks (CNNs) can efficiently capture nonlinear local features of operating voltage and current. The bidirectional long short-term memory network (BiLSTM) is adept at extracting time series features from sequential data. Furthermore, the output features of the BiLSTM layer are input into the attention layer, and the importance of temporal information is evaluated through a weighting method to reduce the interference of irrelevant information on the prediction of wire harness aging indicators. This article collects a large amount of actual data through experiments to quantitatively analyze the physical and chemical changes during the aging process of 12V low-voltage automotive wire harnesses. The experimental design and data acquisition are based on a 12V DC system. By comparing and verifying with other prediction models, this study aims to predict the aging status of wire harnesses through real-time system operation data, achieving early identification and quantitative evaluation of aging.

## 2. Establishment of Wiring Harness Aging Model

Under the influence of electricity and heat during normal operation, the insulation performance of the wiring harness insulation layer will decrease. The insulation aging phenomenon of automotive wiring harnesses includes a decrease in insulation resistance, an increase in insulation dielectric loss, and an increase in temperature during operation.

In order to investigate the aging degree of automotive wiring harnesses, it is necessary to have quantifiable physical quantities to represent their aging degree. The traditional research on cable aging characteristics is to prepare insulation material samples in the laboratory and study their accelerated aging characteristics, such as obtaining physical and chemical properties through fracture elongation, infrared spectroscopy, differential thermal analysis tests, or analyzing the changes in electrical performance through tests such as power frequency dielectric loss factor and breakdown voltage.

Compared to traditional industrial cables, the size of automotive wiring harnesses is much smaller, making them more suitable for testing as a whole. The aging of the entire wiring harness can better simulate the working conditions in practical applications, including not only insulation materials but also the aging of internal copper wires and connection ends, which more comprehensively reflects the aging status of the wiring harness. Therefore, this article adopted high-temperature accelerated aging of complete automotive wiring harnesses instead of making samples for aging testing, and selected non-destructive dielectric loss factor testing to quantify the degree of aging of automotive wiring harnesses.

### 2.1. Dielectric Loss and Aging Degree

The main signals that can be collected inside a car are voltage, current, and environmental temperature and humidity. Therefore, the physical quantity that can determine the degree of aging needs to have a strong correlation with electrical performance, so dielectric loss is chosen.

**Figure 1:**
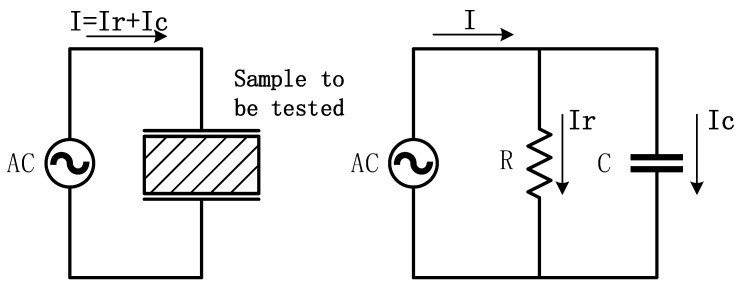
Basic principle of dielectric loss factor testing.

The definition of dielectric loss is the energy loss caused by the hysteresis effect of dielectric conductivity and polarization in an insulating material under the action of an electric field, which refers to the ratio of active power to reactive power of the measured sample. The test principle is shown in Figure 1.

**Figure 2:**
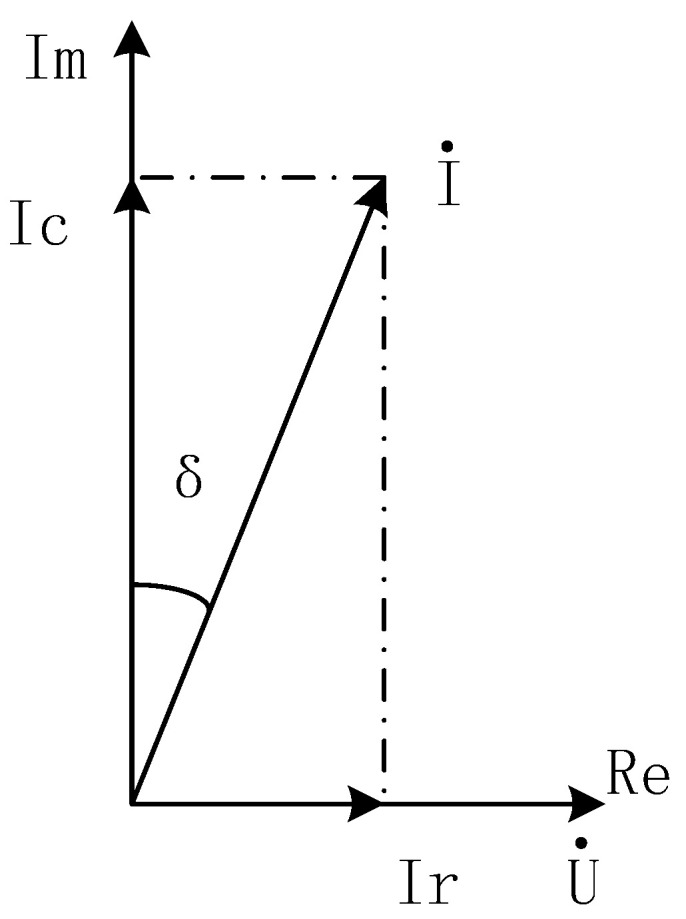
Current vector method.

In capacitive media, current I can be decomposed into capacitive current Ic and resistive current Ir, as shown in Figure 2, which is also a current vector method for calculating dielectric loss factor, also known as dielectric loss tangent, as shown in Figure 2.

According to the definition of dielectric loss and the current vector decomposition method, the calculation (Formula (1)) for the tangent value of dielectric loss can be obtained as follows:

(1) $$tan\delta =\frac{P}{Q}=\frac{U\cdot I_{r}}{U\cdot I_{c}}=\frac{I_{r}}{I_{c}}\times 100\%,$$

Dielectric loss reflects the insulation performance of wire harness insulation materials. The greater the dielectric loss, the more electrical energy the material converts into thermal energy, resulting in increased energy loss.

### 2.2. Accelerated Aging Experiment

Due to the slow changes in the wiring harness under natural working conditions, this article designs an accelerated aging experiment with the aim of quickly generating samples covering the entire aging process from the initial state to severe aging while simulating the insulation aging mechanism of the wiring harness as much as possible, ensuring that the collected dielectric loss data are representative and comprehensive. And accelerated aging experiments can be conducted under controlled laboratory conditions to maintain consistency and reproducibility of the experiment, thereby improving the accuracy and reliability of the data.

This study selected 140 °C (close to the upper limit of the standard) as the experimental temperature, aiming to more efficiently simulate the accelerated aging process under extreme operating conditions. A preliminary experimental analysis revealed that under 140 °C conditions, the dielectric loss of the wiring harness exhibited a significant and distinguishable increasing trend with prolonged aging time, effectively covering the entire lifecycle from the initial state (0 h) to severe aging (720 h). In comparison, using 120 °C would require extending the experimental duration to several thousand hours, while a higher temperature such as 160 °C could lead to rapid embrittlement of the insulation layer, thereby compromising the continuity of experimental data.

The main method used in this experiment is high-temperature aging of the entire wire harness at 140 °C, with 72 h as a cycle. A batch of wire harnesses are taken as test samples in sequence, from 72 h, 144 h, and 216 h up to 720 h, to obtain wire harness samples with different aging durations. Data are collected and analyzed.

### 2.3. Feature Selection and Model Input

When selecting physical quantities for evaluating the aging degree of insulation materials in wire harnesses, electrical performance indicators are particularly important due to their direct correlation with the material state. In the automotive environment, voltage and current signals are the easiest electrical parameters to obtain. They can provide direct information about the insulation status of the wiring harness and may also be used by the system itself to detect whether there is overvoltage in the circuit, making it easier to obtain this value. Therefore, this study considers voltage and current as key input feature quantities for the neural network model.

In the research on cable insulation material aging, the temperature effect is particularly critical. The Arrhenius equation is commonly used as the cornerstone for describing the relationship between temperature and reaction rate, providing a mathematical model for quantifying this effect. The equation is expressed as follows:

(2) $$k=A\times e^{-\frac{b}{T}}$$

where *k* represents the reaction rate constant, which is directly related to the rate of material aging process; *A* and *b* are constants related to insulating materials; and *T* is the absolute temperature, measured in Kelvin, directly involved in the exponential term of reaction rate, highlighting the exponential effect of temperature on reaction rate.

Given the significant role of temperature in the aging process of materials, and considering that it is not constant in practical applications, this study chooses temperature as a key feature quantity to model and predict the aging behavior of materials through neural network algorithms, in order to reveal its complex interaction with the aging rate of materials.

Based on the derived feature quantities of voltage and current, in order to further enhance the recognition ability of neural network models for the aging state of wire harness insulation materials, in addition to the basic voltage, current, and temperature feature quantities, this study also considers a series of derived feature quantities based on voltage and current. These derived feature quantities can provide richer information, help reveal the complex relationship between voltage and current during the aging process, and improve the fitting performance of the model.

The product of voltage and current (P) is associated with the power loss in the wiring harness to capture information on the increase in power loss caused by the increase in wiring harness resistance during the aging process. The impedance size of the circuit is used to identify impedance changes caused by wire harness aging. The square of the current (I^2^) is directly related to the heat loss in the wire harness, because the heat loss is proportional to the square of the current, and the change in the square of the current will be more sensitive to capture more detailed change information. Excluding the voltage drop of the load, the voltage value in this feature is more closely related to the voltage drop information of the harness itself, which can more accurately identify the aging information of the harness.

## 3. System Architecture

### 3.1. Hardware Platform

The hardware platform mainly simulates the low-voltage electrical system circuit of the 12V resistive load in the vehicle, such as the wiring harness research for loads such as car lights and seat heating. Connect the intelligent fuse module with a 12V power supply, and then connect the wiring harness to the resistive load to construct a complete closed circuit.

The intelligent fuse module, as the core part of the entire system, not only includes electronic fuses for overload protection, but also is equipped with a voltage acquisition module. Among them, the voltage acquisition module works using a voltage division method. By setting a voltage division resistor network, the higher voltage of the wire harness and the load part is divided by a certain proportion, and then high-precision voltage sensors are used for precise measurement to obtain accurate voltage signals. The current acquisition module uses the shunt method to achieve this. By connecting a low resistance shunt resistor in series in the circuit, a voltage drop proportional to the current is generated at both ends. The voltage drop is detected by a current sensor, and the actual current value is converted according to Ohm’s law to collect the current signal of the entire closed circuit. In addition, the temperature acquisition module uses temperature sensors to sense the ambient temperature of the system’s operation. The sensors convert the temperature signal into corresponding electrical signals, achieving the real-time monitoring of temperature for subsequent data analysis and neural network training.

### 3.2. Software Scheme

**Figure 3:**
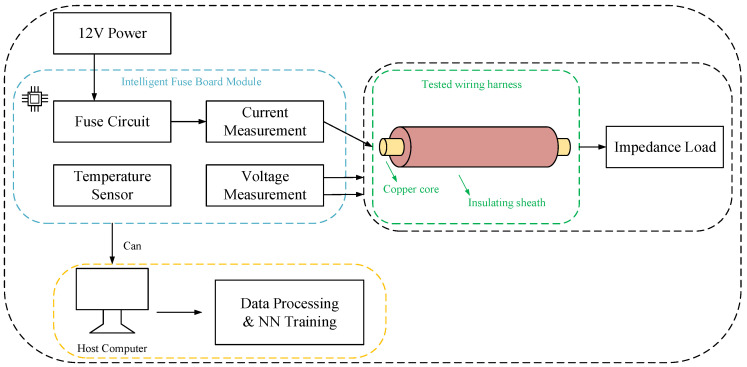
Overall structure of the measurement system.

In terms of software, the voltage, current, and temperature data collected by the intelligent fuse module are converted from analog to digital and transmitted to the PC upper computer through the CAN-to-USB interface. Perform data cleaning, normalization processing, remove outliers and noise, and improve data quality. The processed data are used for neural network training. The overall structure is shown in Figure 3.

## 4. Methods

### 4.1. CNN-BiLSTM-Attention Algorithm

#### 4.1.1. Convolutional Feature Extraction Layer

Based on the local perceptual characteristics of convolutional kernels, one-dimensional convolutional layers are used for the multi-scale mining of degraded features [18,19]. Extract local pattern features of device monitoring data through the convolution operation shown in Equation (3).

(3) $$h^{\left(l\right)}=\sigma \left(W^{\left(l\right)}*h^{\left(l-1\right)}+b^{\left(l-1\right)}\right)$$

where $W^{\left(l\right)}$ is the learnable convolution kernel parameter, * represents the convolution operation, and σ is the ReLU activation function. By stacking convolutional layers and max pooling layers, a hierarchical feature representation is constructed, effectively overcoming the limitations of traditional manual feature engineering.

#### 4.1.2. BiLSTM Neural Network

**Figure 4:**
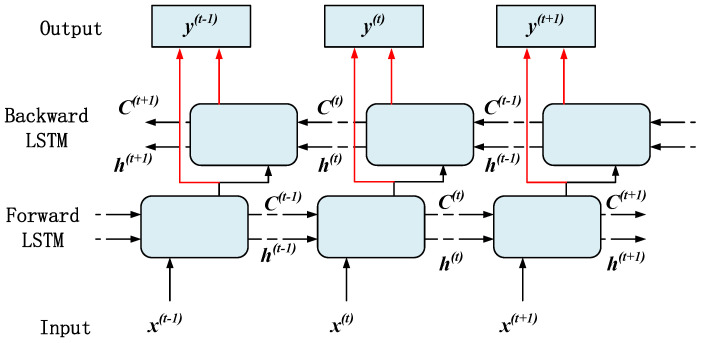
Schematic diagram of BiLSTM network structure.

The bidirectional long short-term memory network (BiLSTM) selected in this article is a variant of LSTM. It processes sequence data by introducing two LSTM layers, capturing the forward and backward dependencies, and fusing the hidden layer states through weight coefficients [20,21,22], providing a more comprehensive data perspective. Its unit structure is shown in Figure 4.

The final output is a combination of forward and backward LSTM outputs, so that BiLSTM can use the information of the entire sequence to make decisions. The calculation process is shown in Equations (4)–(6).

(4) $${\vec{h}}_{f}\left(t\right)=LSTM(x^{\left(t\right)},{\vec{h}}_{f}\left(t-1\right))$$

(5) $${\overset{\leftarrow }{h}}_{b}\left(t\right)=LSTM(x^{\left(t\right)},{\overset{\leftarrow }{h}}_{b}\left(t-1\right))$$

(6) $$y^{\left(t\right)}=W_{f}{\vec{h}}_{f}\left(t\right)+W_{b}{\overset{\leftarrow }{h}}_{b}\left(t\right)+b$$

where ${\vec{h}}_{f}\left(t\right)$ is the hidden layer state of forward propagation, $x^{\left(t\right)}$ is the input, ${\overset{\leftarrow }{h}}_{b}\left(t\right)$ is the hidden layer state of backward propagation, $W_{f}$ and $W_{b}$ are the weight matrices corresponding to each propagation direction, and b is the bias optimization parameter.

#### 4.1.3. Attention Layer

In this architecture, the $h_{t}$ output from the hidden layer of the BiLSTM layer is used as the input for the attention layer [23]. The importance level of each historical time step t is calculated through additive attention, which evaluates the contribution of each time step in the sequence to the current task [24]. Then, score it using the softmax function, and the score calculation formula is as follows:

(7) $$s\left(h_{i}\right)=vtanh\left(Wh_{k}+Uh_{i}+b\right)$$

(8) $$\alpha_{k}=softmax\left(s\left(h_{i}\right)\right)$$

where $s\left(h_{i}\right)$ is the similarity score, and v is the weight vector used to calculate the similarity score; *W* and *U* are the similarity score weight matrices for time *k* and time *i*, respectively; and $\alpha_{k}$ is the calculated attention weight value.

Finally, by subtracting each historical state output from its corresponding attention weight and summing them up, the optimized output result h is obtained:

(9) $$h=\sum_{t=1}^{k}a_{t}h_{t}$$

where $h_{t}$ is the input at time *t*, and $a_{t}$ is the weight value corresponding to time *t*.

#### 4.1.4. Overall Architecture of CNN-BiLSTM-Attention

**Figure 5:**
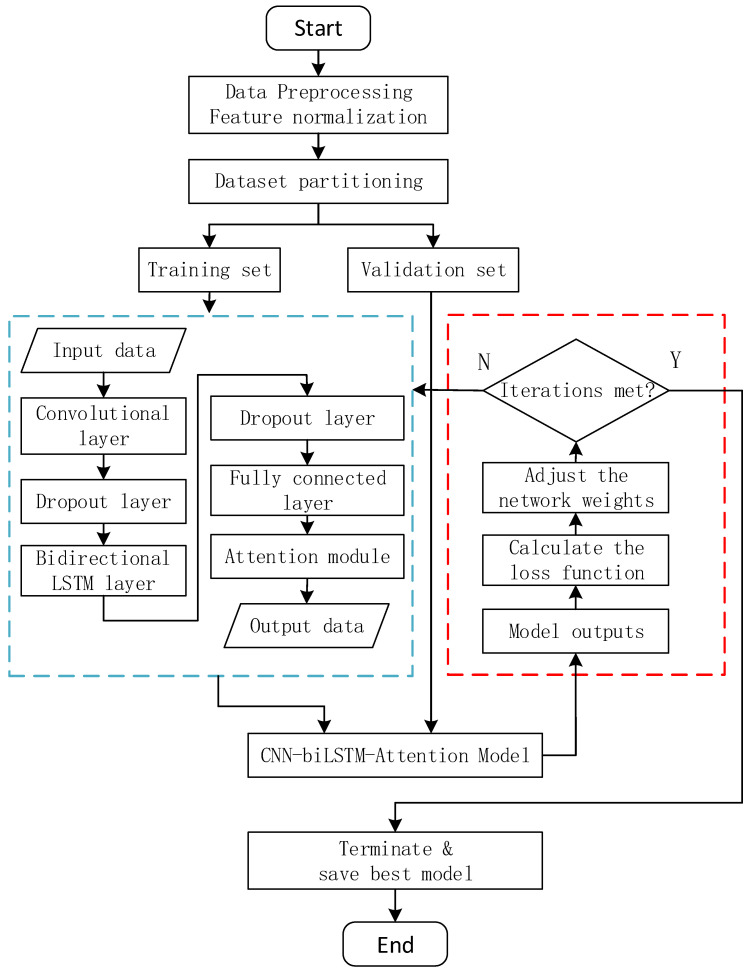
Algorithm flowchart.

This article constructs a CNN-BiLSTM-Attention hybrid model, which captures local spatial features through convolutional neural networks, models temporal dynamic evolution through bidirectional LSTM, and combines attention mechanisms to achieve feature adaptive weighting. The overall framework is shown in Figure 5.

Prior to model construction, raw data undergo systematic processing: temporal features are extracted through data cleaning (e.g., handling missing values and outliers), followed by Min–Max normalization to eliminate feature scale discrepancies. The dataset is then split into training and validation sets while preserving temporal continuity. The input data are fed into the model, which sequentially consists of a convolutional layer (Conv1D, kernel size 1, 64 filters), a first dropout layer (rate 0.3) for random neuron deactivation to mitigate overfitting, a bidirectional LSTM layer (BiLSTM, 64 units per direction, a second dropout layer (rate 0.3) for enhanced regularization, a fully connected layer for nonlinear feature transformation, an attention mechanism to dynamically weight critical time steps, and finally a Sigmoid output layer for prediction.

### 4.2. Data Acquisition and Processing

#### 4.2.1. Feature Value Selection

This article uses 8 numerical values or derived values collected through electronic fuse circuits as input features for the model. In the accelerated aging experiment, each group has 6 wires of the same aging duration, with 5 wires taken from each group as the training set data and 1 wire as the test set data. Each wire harness is connected to the electronic fuse circuit, and data such as timestamp, voltage, current, temperature, etc., are collected and saved as raw data with a sampling period of 100 ms. The sliding window of LSTM is selected as 300, and a single-step prediction strategy is adopted. These 300 data points are used as a time series as the input of the model to predict the current aging state of this wire harness, which is the quantitative indicator of dielectric loss selected in this paper. While humidity was not experimentally controlled in this study, its potential effect on dielectric loss can be inferred from prior work [15]. Moisture absorption in insulation materials may accelerate ionic conduction, leading to higher dielectric loss. Future studies should incorporate humidity sensors to quantify its interaction with temperature and aging. Despite this limitation, the current model’s generalizability is supported by controlled temperature conditions, which dominate the Arrhenius-based aging mechanism Equation (10). The complete feature data and descripition are presented in Table 1.

#### 4.2.2. Model Evaluation Indicators

This article uses two evaluation metrics, mean square error (MSE) and mean absolute error (MAE), to evaluate the performance of aging prediction models. The smaller the values of MSE and MAE, the more accurate the prediction of the aging degree of the wiring harness. The calculation formulas for these two indicators are shown in Equations (10) and (11).

(10) $$\mathrm{MSE}\;=\frac{1}{n}\overset{n}{\underset{i=1}{\sum }}{\left(y\left(i\right)-\;y^{\prime }\left(i\right)\right)}^{2}$$

(11) $$\mathrm{MAE}=\frac{1}{n}\overset{n}{\underset{i=1}{\sum }}\left|y\left(i\right)-\;y^{\prime }\left(i\right)\right|$$

where n is the number of predicted samples, y^′ (i) is the predicted value of the model, and y(i) is the true label value.

## 5. Results and Discussion

**Figure 6:**
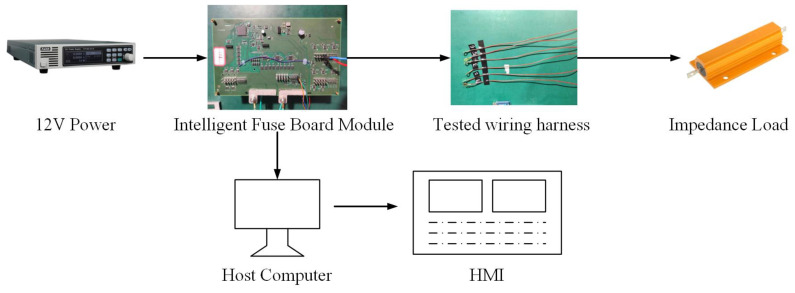
Experimental platform.

To validate the measurement effectiveness of the system, we established an experimental platform (Figure 6) centered around a TC367 microcontroller serving as the core control unit for synchronized data acquisition. The main sensor comprises current sensors (ACS712ELCTR-20A) and digital temperature sensors (DS18B20-MS). Real-time data streams were transmitted via a CAN bus to custom-developed host computer software for monitoring and analysis. This platform enabled two-stage validation: initial data collection during the development phase followed by real-time model performance validation.

### 5.1. Experimental Results of Dielectric Loss

This experiment collected data from 11 groups of 66 wire harnesses, ranging from brand new wire harnesses to 720 h of high-temperature aging. Each wire harness was sampled at a frequency of 10 Hz for 10 min to collect approximately 8000 sets of voltage, current, and other data. In this study, one wire harness from each group was selected as the test set to verify the predictive performance of the trained model.

**Figure 7:**
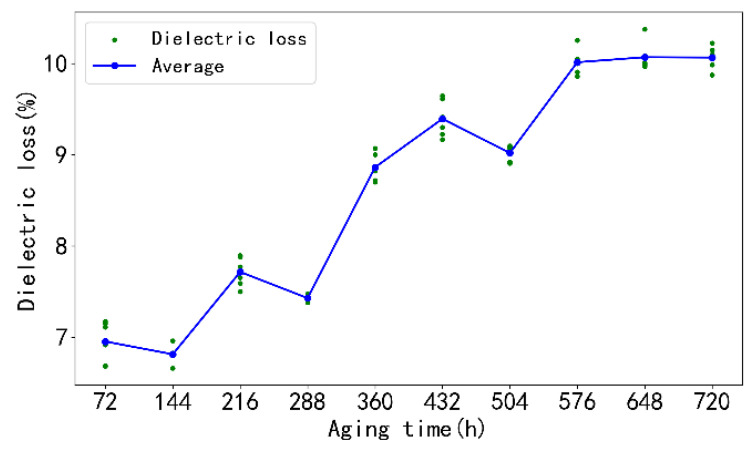
Trend diagram of the relationship between harness dielectric loss and aging time.

The results of the high-temperature accelerated aging experiment are shown in Figure 7. With the extension of aging time, the dielectric loss value shows a certain increasing trend. This phenomenon is due to the accelerated molecular motion inside the material caused by the high-temperature environment, resulting in an increase in the polarity of the material and thus an increase in the dielectric loss. In addition, the high temperature promotes chemical decomposition or cross-linking reactions inside the material, which may generate more charge traps or defects, thereby increasing the dielectric loss of the material.

Between 72 and 288 h in the early stage of aging, the increase in dielectric loss value is relatively slow, and the wire harness has good adaptability to high-temperature environments in the initial stage. As the aging process continues, the growth rate of dielectric loss value accelerates after 288 h, and the cumulative effect of internal damage in the material begins to emerge. After 576 h, the dielectric loss value tends to stabilize, and the insulation material has reached a new equilibrium state.

From the experimental results of dielectric loss testing, it can be seen that the actual dielectric loss value shows a positive correlation trend with the increase in aging time, but this trend is not completely linear or exponential, but there is a rough interval relationship that can represent different degrees of aging of the wire harness. For example, if the aging time is 288 h or less, the dielectric loss is mostly below 8%; if the aging time is 288 to 504 h, the dielectric loss value is between 8% and 9.5%; and if the aging time is over 576 h, the dielectric loss values are all greater than 9.5%.

### 5.2. Analysis of Neural Network Prediction Results

In order to better validate the performance of the CNN-Bilstm-Attention neural network, LSTM neural network model and Random Forest machine learning models were selected for comparison, and the MSE and MAE values of each model were calculated for comparison. The model was trained with a learning rate of 0.001 using the Adam optimizer, with a batch size of 128 and 300 epochs. Early stopping was applied with a patience of 15 epochs to prevent overfitting, monitoring the validation loss.

According to the data in Table 2, it can be seen that considering both MSE and MAE indicators, the CNN-Bilstm-Attention model exhibits the lowest error values. To validate the model’s stability across diverse experimental settings, we conducted rigorous evaluations through 10 independent runs, observing consistently low performance variations (MSE = 0.00292 ± 0.0004, MAE = 0.02806 ± 0.003). The observed results primarily stem from the efficient synergy of the multi-layer structure: the CNN extracts spatial features through local perception, enhancing the model’s stable capture of key patterns in input data; BiLSTM’s bidirectional temporal modeling effectively balances the dependencies in sequential data, reducing sensitivity to local fluctuations; and the attention mechanism dynamically allocates feature weights, suppressing noise and boosting the contribution of important temporal nodes. This progressive architecture enables the model to grasp the inherent data patterns more consistently across experiments. As a result, it maintains high prediction accuracy while significantly reducing the volatility of evaluation metrics, thus improving the model’s robustness.

This indicates that the model not only has a small average error in predicting wire harness aging, but also has low sensitivity to outliers, providing more stable and reliable prediction results.

In order to more intuitively observe the performance differences among the three models, a set of samples was selected to draw a line graph of the predicted values under each model.

**Figure 8:**
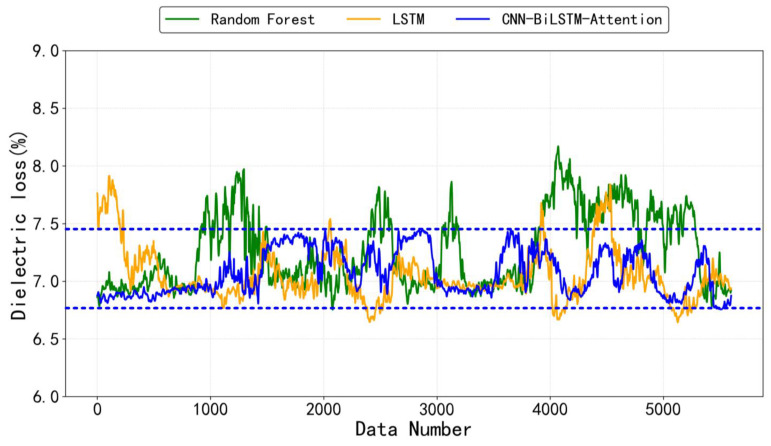
Comparison of three models’ predicted values in line charts.

Figure 8 show the predicted values analysis of the same test sample wire harness, with an actual dielectric loss value of 7.17%. Among the continuously predicted values of about 5000 points, the CNN-BiLSTM-Attention model’s predicted values are more convergent to the actual values than other models, with a smaller fluctuation range. This indicates that the model can more accurately capture the complex features and dynamic changes in data, while the predicted points of LSTM and Random Forest models show greater dispersion. Further verification shows that the CNN-BiLSTM-Attention model outperforms the other two models in evaluation metrics such as mean square error (MSE) and mean absolute error (MAE).

The practical implications of the predicted aging levels can be further translated into actionable strategies for vehicle maintenance and safety systems. Based on the observed dielectric loss thresholds (e.g., <8% for early aging, 8–9.5% for moderate aging, and >9.5% for severe aging), targeted interventions can be designed. For instance, when the dielectric loss exceeds 8%, the vehicle’s onboard diagnostics system could trigger a warning signal to recommend inspection or reduce power output in high-load circuits, thereby mitigating further degradation. At thresholds above 9.5%, proactive replacement of the harness could be prioritized to avoid critical failures.

Integration with existing vehicle diagnostic platforms is feasible through real-time data streaming from the intelligent fuse module to the central control unit. By embedding the trained CNN-BiLSTM-Attention model into edge computing devices, continuous monitoring and instant aging predictions can be achieved without compromising system latency.

## 6. Conclusions

In this study, the CNN-BiLSTM-Attention neural network model was successfully applied to predict the aging degree of automotive wiring harnesses. By analyzing the wiring harness data from brand new to high-temperature aging for 720 h, we found a positive correlation between dielectric loss and aging time, and this relationship exhibits different characteristics at different aging stages. Our experimental results indicate that the CNN BiLSTM Attention model has superior performance in predicting the degree of wire harness aging. Compared with traditional LSTM models and Random Forest machine learning models, it exhibits lower error values in both MSE and MAE evaluation metrics, demonstrating better prediction accuracy and stability.

The remaining prediction errors in the final results may originate from the following aspects: despite data cleaning and normalization processes, sensor sampling noise and transient circuit interference might still affect the quality of input features; the absence of environmental variables such as humidity may result in incomplete modeling of certain aging mechanisms; and the current model, trained solely on aging data at a fixed temperature (140 °C), lacks coverage of dynamic temperature variations in real operational conditions, potentially affecting prediction stability.

Future work will involve multi-temperature joint experiments and the integration of humidity sensors to further optimize the model. Based on the predicted degree of aging, we can develop intelligent control strategies to extend the service life of the wiring harness. For example, when it is predicted that the wiring harness is about to enter the accelerated aging stage, the aging process can be slowed down by reducing the output power or adjusting the working mode to reduce heat loss. The model and method of this study can be applied to aging prediction problems in other fields, such as connectors connected to wiring harnesses and various loads, to verify the universality and effectiveness of the model.

## Figures and Tables

**Table 1:** Feature data collected in the electronic fuse circuit.

Feature	Description (Unit)
Voltage	Circuit voltage (V)
Current	Circuit current (A)
Tem	Operating ambient temperature (°C)
Power	Power (W)
Impedance	Circuit impedance magnitude (Ω)
circuit_voltage	Voltage excluding load drop (V)
current_squared	Squared current (A^2^)
sqrt_current_voltage	Square root of (voltage^2^ + current^2^)

**Table 2:** Comparison of model performance.

Model	MSE	MAE
Random Forest	0.00758	0.07397
LSTM	0.00469	0.04157
CNN-BiLSTM-Attention	0.00292	0.02806

## Data Availability

The raw data supporting the conclusions of this article will be made available by the authors on request.
